# Fetal Amelia With Hypoplastic Tibia and Terminal Fibular Hemimelia: A Case Report With Review of the Literature

**DOI:** 10.7759/cureus.32849

**Published:** 2022-12-22

**Authors:** Vrushali Dalvi, Avinash Dhok, Prashant Onkar, Kajal Mitra, Pooja Ladke

**Affiliations:** 1 Department of Radiodiagnosis, NKP Salve Institute of Medical Sciences and Research Center & Lata Mangeshkar Hospital, Nagpur, IND

**Keywords:** longitudinal and transverse deficiency of limb, developmental anomaly, congenital limb deformity, hemimelia, amelia

## Abstract

Congenital limb deformities, with a birth frequency of 0.55 per 1,000, are extremely rare prenatal defects that can present with either partial or complete lack of a limb or a specific portion of a limb. Amelia is a sporadic anomaly that is defined by the complete absence of a limb's skeletal elements, whereas hypomelia is defined by the incomplete development of a limb's skeletal elements. We present the case of a neonate with gross facial deformities in the form of the absence of both external ears and a saddle-shaped nose. The absence of the right lower limb bud was seen. The left lower limb was underdeveloped, noted only up to the thigh region with the hypoplastic distal part of the leg and absent foot. Genitals and the anus were absent. To the best of our knowledge, this case is exceptional in that congenital limb abnormalities are present at birth along with accompanying genital underdevelopment.

## Introduction

Congenital limb deformities affect 0.55 per 1000 babies born [1]. Congenital limb deformities are extremely rare prenatal anomalies that can be present either as a partial and complete absence of a limb or a specific segment of a limb. Amelia is a sporadic anomaly that is characterized by the complete absence of a limb's skeletal elements, whereas hypomelia is characterized by the incomplete development of a limb's skeletal elements [2]. Amelia is a condition that affects approximately 0.05 to 0.09 out of every 10,000 newborn babies [2,3]. They are either presented with multiorgan malformations, especially renal and abdominal wall anomalies, or can present as an isolated defect [4-6]. This severe limb insufficiency has been associated with numerous factors, including teratogenic drugs like thalidomide or in some cases alcohol consumption. Also, vascular insufficiency is caused by factors such as amniotic bands syndrome and diabetes in pregnant females [7-9]. Amelia's genesis has been related to various types of heredity, including the autosomal dominant, autosomal recessive, and X-linked dominant modes of inheritance, indicating this condition's genetic heterogeneity [10]. These deformities have a very low risk of recurrence. In order to properly educate parents who have fetal malformations, prenatal evaluation, including in-depth ultrasound and amniocentesis, is essential. Prior studies have reported amelia associated with several birth defects identical to Robert syndrome and Schinzel phocomelia [11]. Nowadays, reports of infants with severe facial deformities, tetra-amelia syndrome, and pulmonary abnormalities are reported despite normal chromosomal findings. Hence, a prenatal diagnosis that includes in-depth ultrasound and amniocentesis has a crucial role in counseling parents with fetal anomalies [11].

## Case presentation

Patient information

The mother was a 30-year-old Gravida 1 Para 1 Abortus 0 Living 0 woman in good health and arrived for her visit to our institution at 20 weeks of gestation. There was no consanguinity or significant family history, and the father was a 32-year-old healthy man. No known teratogenic exposure occurred during pregnancy.

Clinical findings

**Figure 1:**
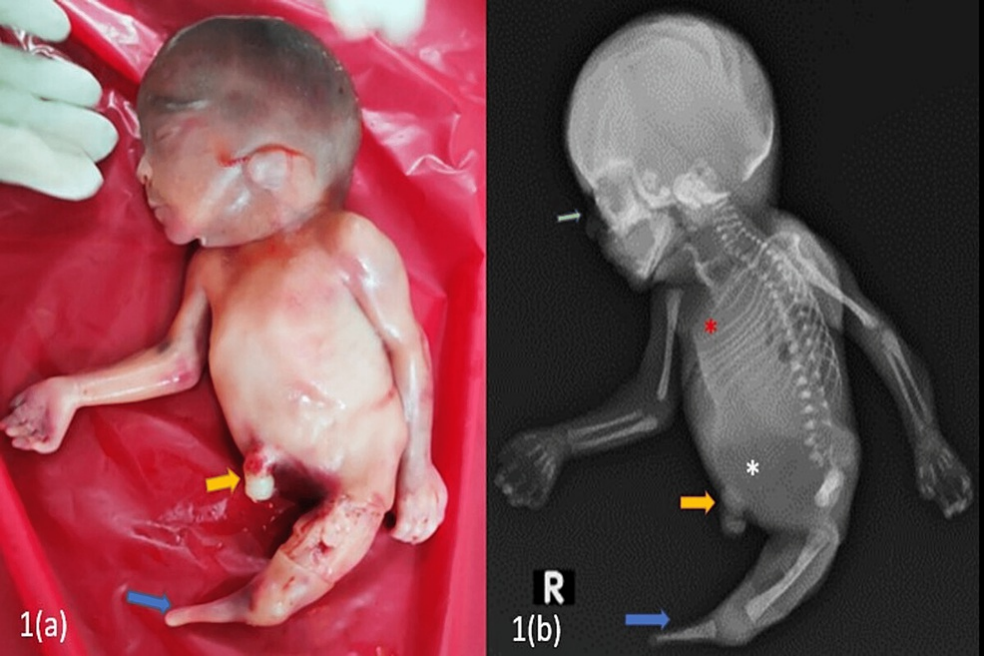
(a) Clinical image showing the complete absence of the right lower bud (yellow arrow) and hypoplastic/dysplastic left limb bud (blue arrow). (b): Babygram showing amelia (complete absence) of the right lower limb (yellow arrow), hypoplastic/dysplastic tibia with complete absence of the left fibula and left foot (blue arrow), hypoplastic left iliac bone with the absence of the entire right pelvis, left ischium, and left pubic rami and hypoplastic/dysplastic lower lumbar vertebrae (white asterisk), crowding of ribs (red asterisk)

**Figure 2:**
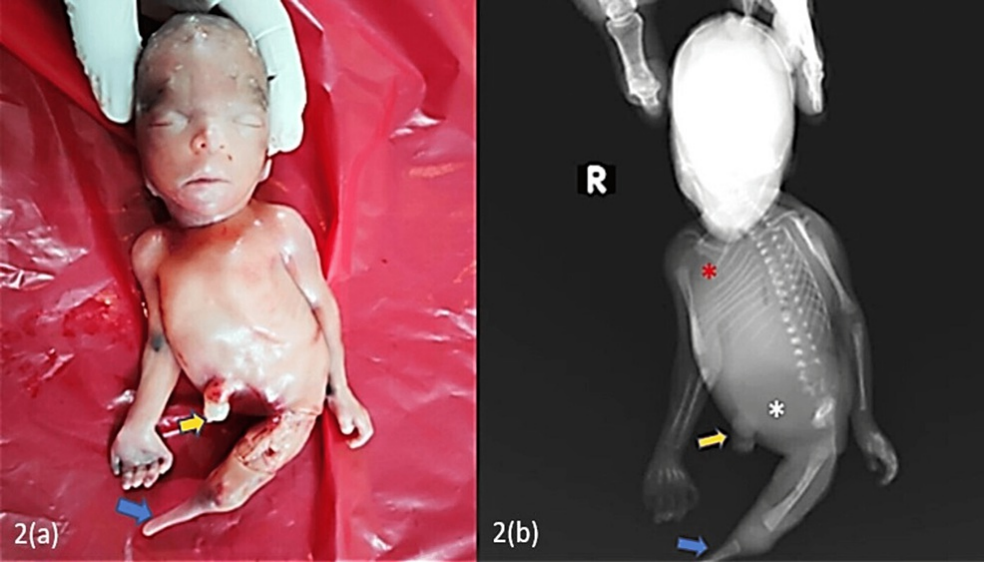
(a) Clinical image showing the complete absence of the right lower bud (yellow arrow) and hypoplastic/dysplastic left limb bud (blue arrow). (b): Babygram showing amelia (complete absence) of the right lower limb (yellow arrow), hypoplastic/dysplastic tibia with the complete absence of the left fibula and left foot (blue arrow), hypoplastic left iliac bone with the absence of the entire right pelvis, left ischium, and left pubic rami and hypoplastic/dysplastic lower lumbar vertebrae (white asterisk), crowding of the ribs (red asterisk)

**Figure 3:**
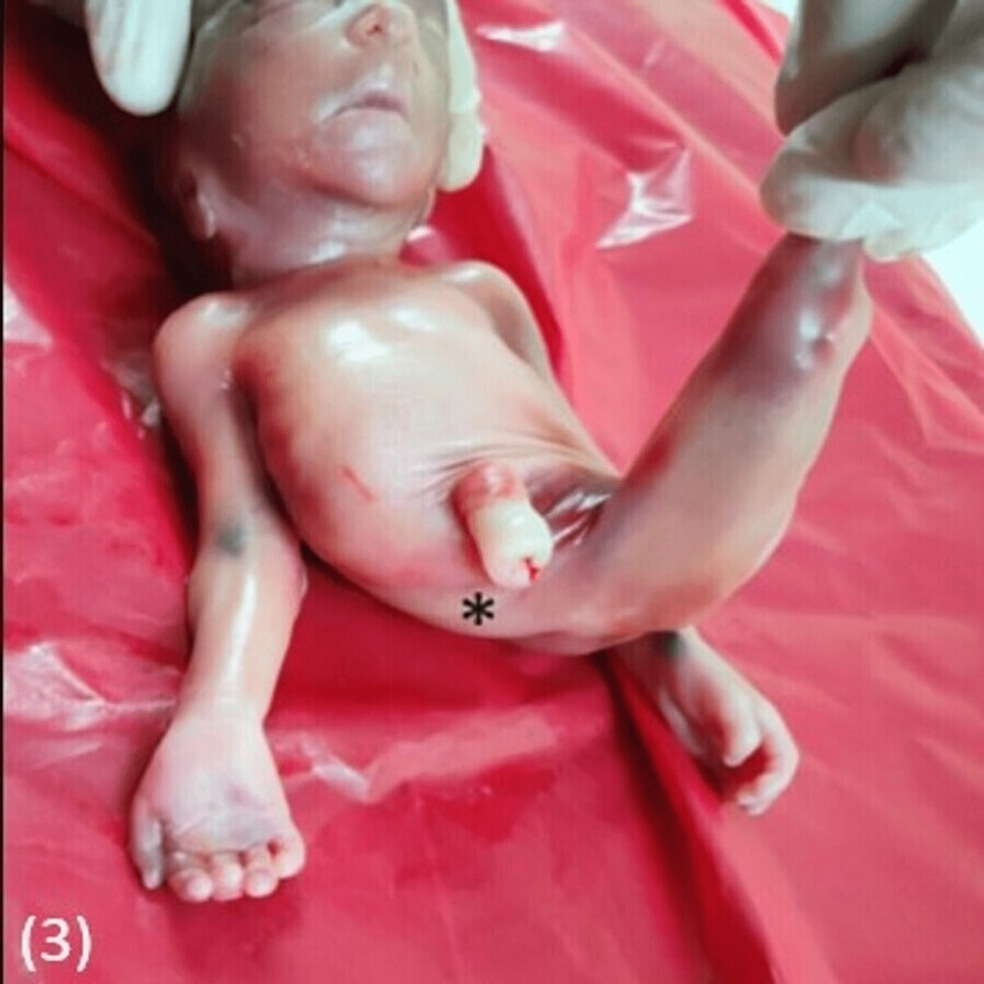
Clinical image showing absent genitalia and anus (asterisk)

On antenatal ultrasonography done at a private center outside our institute, the right lower limb was completely absent and the left lower limb was hypoplastic. Although bilateral renal agenesis was observed, the remainder of the infant appeared normal. The poor prognosis was conveyed to the couple. They opted to terminate the pregnancy medically at 20 weeks due to fetal indication at our institute. On examination, gross facial deformities were discovered in the form of the absence of both external ears and a saddle-shaped nose in the baby. The absence of the right lower limb bud was seen. The left lower limb was underdeveloped, noted only up to the thigh region with the hypoplastic distal part of the leg and absent foot (Figures 1, 2). Also, the genitals and the anus were absent (Figure 3).

A babygram was performed after birth, which showed amelia (complete absence) of the right lower limb, left-sided terminal fibular hemimelia (complete absence of the left fibula) with hypoplastic/dysplastic tibia, and complete absence of the left foot (Figures 1, 2). Hypoplastic left iliac bone with the absence of the entire right pelvis, left ischium, and left pubic rami. Lower lumbar vertebrae appeared hypoplastic/dysplastic. Crowding of ribs was noted on the right side. The absence of nasal bone was noted (Figures 1, 2).

Diagnosis

The diagnosis of congenital limb deformity in the form of fetal amelia with hypoplastic tibia and terminal fibular hemimelia was considered in view of imaging findings.

## Discussion

A newborn with hypomelia of the left limb and amelia of the right limb, absent genitalia, and anus are included in this report. We are reporting this case because of the unusual rarity of this congenital defect. Amelia was previously considered to be a rare abnormality with a low chance of recurrence or evidence of genetic causation. However, the autosomal dominant, autosomal recessive, and X-linked dominant modes of inheritance, all have been implicated in the etiology of Amelia, reflecting this condition's genetic variation [10].

This report discusses the amelia that was discovered after a 20-week prenatal scan in a non-consanguineous couple's pregnancy. The findings of the clinical evaluation confirmed those of the fetal findings.

Nowadays, fetuses with gross facial deformities, tetra-amelia syndrome, and multiorgan abnormalities are now being reported in the literature despite having normal chromosomal findings [11].

Song et al. have previously described various abnormalities in the form of amelia, which resembles Robert syndrome an autosomal recessive condition that presents as a variety of morphologic defects [11].

Except for bilateral renal dysgenesis, no other significant defects were found during the prenatal ultrasonography in the present patient. Infants presenting with such morphologic defects have a poorer prognosis while most neonates with associated single or multiorgan abnormalities succumb to death early in life, within a year. The results of the autopsy would have been useful, but the parents refused to allow for a pathological examination and chromosomal analysis. Because there hasn't been an autopsy on this patient, it's difficult to categorize the condition precisely. Only a few families have evidence of amelia's probable recurrence [9,12].

Few classifications were found in the literature. Out of these, we found the Centers for Disease Control and Prevention (CDC) nomenclature for limb defects useful. According to this, there are two main categories of limb deficiencies: longitudinal and transverse. The following subgroups of longitudinal deficiencies are identified along the long axis of the limb: preaxial (radial and tibial side), postaxial (ulnar and fibular side), and axial (central). Transverse deficits, on the other hand, occur across (transversally to) the limb's long axis. Transverse deficiencies can either be terminal (more common), in which case the terminal component of the limb is completely absent, or intercalary, in which case the terminal part of the limb is partially absent but still present, although deformed. As limb reduction abnormalities own a tendency to have different etiologies and pathology depending on their specific subtype, it is critical to classify them correctly [13].

Regarding genetic or teratogenic causes, in this case, maternal antenatal and family history were not contributing factors, and it doesn't seem likely that maternal infection was the cause. Understanding the etiology of this case further is challenging. A low recurrence rate was discussed with these parents, and they were counseled to undergo an anomaly scan sooner in future pregnancies.

## Conclusions

In order to properly counsel parents with fetal anomalies, prenatal assessment, including in-depth ultrasound and amniocentesis, is crucial. For the purpose of determining the demographic characteristics, etiology, risk factors, and associations of all forms of limb defects, a congenital deformities surveillance and record system must be created. Also, the development of a systematic and detailed classification system that includes a wider range of limb anomalies is needed.
